# Validity and psychometric characteristics of the psoriatic arthritis quality of life (PSAQoL) questionnaire in the Turkish population

**DOI:** 10.1007/s00296-025-05911-6

**Published:** 2025-06-16

**Authors:** Mehmet Tuncay Duruöz, Kemal Nas, Sevtap Acer Kasman, Emre Uzun, Halise Hande Gezer

**Affiliations:** 1 Rheumatology Division, Physical Medicine and Rehabilitation Department, Marmara University School of Medicine, Istanbul, Turkey; 2 Physical Medicine and Rehabilitation Department, Sakarya University School of Medicine, Immunology Subdivision, Sakarya, Turkey; 3 Rheumatology Division, Physical Medicine and Rehabilitation Department, Bahçeşehir University School of Medicine, Istanbul, Turkey; 4 Physical Medicine and Rehabilitation, Sakarya Training and Research Hospital, Sakarya, Turkey

**Keywords:** Psoriatic arthritis, Quality of life, Patient reported outcome measures, Validation study, Reliability, Psychometrics, Cross-cultural comparison, Questionnaires

## Abstract

The aim of this study was to translate and culturally validate the Psoriatic Arthritis Quality of Life Questionnaire (PsAQoL) into Turkish and to evaluate its reliability and validity in patients with psoriatic arthritis (PsA). A total of 162 PsA patients diagnosed according to CASPAR criteria were recruited from two rheumatology clinics. The PsAQoL was translated and culturally adapted into Turkish using a standardized forward-backward translation method. Internal consistency was assessed using Cronbach’s alpha. Construct validity was evaluated by correlating PsAQoL scores with quality of life (SF-36), functional (HAQ), emotional (HADS), and clinical disease activity indices (DAPSA, BASDAI, DAS28). The Turkish version of the PsAQoL was found to be clear, concise, and well understood by patients. The average time to complete the questionnaire was 3.3 ± 0.9 min. Internal consistency was good (Cronbach’s α = 0.930). Strong correlations were found with SF-36 physical (*r* = − 0.744) and mental components (*r* = − 0.731), indicating convergent validity. Moderate correlations were observed with HAQ (*r* = 0.533), VAS pain (*r* = 0.408), HADS-Anxiety (*r* = 0.535), and HADS-Depression (*r* = 0.517), while correlations with unrelated clinical parameters such as age and PASI were weak or insignificant, indicating divergent validity. No floor or ceiling effects were detected, and there were no missing responses. The Turkish version of the PsAQoL is a valid, reliable, and practical tool for assessing disease-specific quality of life in PsA. Its ease of use and psychometric strength support its application in both clinical practice and researchs.

**Supplementary Information:**

The online version contains supplementary material available at 10.1007/s00296-025-05911-6.

## Introduction

Psoriatic arthritis (PsA) is a chronic inflammatory arthritis associated with skin and nail psoriasis, which is a type of spondyloarthropathy characterized by axial skeletal involvement, symmetric polyarthritis or asymmetric oligoarthritis of peripheral joints, enthesitis, and rheumatoid factor negativity [1]. PsA affects approximately 0.3–1% of the general population and 5–30% of patients with psoriasis, with an onset typically between the ages of 30 and 55 [2].

In PsA, compared to the general population, a decrease in quality of life has been observed, particularly in the domains of physical function, pain, and role limitations. Quality of life is also adversely affected by comorbidities more commonly seen in PsA than in other rheumatic diseases, such as obesity, metabolic syndrome, cardiovascular disease, depression, and fibromyalgia [3–5]. To assess the quality of life, general indices such as the Short Form-36 (SF-36), Nottingham Health Profile (NHP), and European Quality of Life 5 Dimensions (EQ-5D) are available, but these indices are not disease-specific and don’t capture all aspects of disease. The Psoriatic Arthritis Quality of Life Questionnaire (PsAQoL) is a quality of life index specifically developed for PsA, and unlike other indices, this model includes needs-based quality of life data [6]. According to the GRAPPA–OMERACT, assessing health-related quality of life in PsA is considered essential both for research and routine patient care. In these recommendations, three key suggestions have been emphasized. First, quality of life has been identified as one of the core domains of PsA, and its inclusion as an outcome measure in clinical trials is strongly recommended. Second, GRAPPA–OMERACT supports the use of disease-specific instruments for assessing quality of life, highlighting the PsAQoL as a validated and sensitive tool for this purpose. Third, while generic instruments such as the SF-36 or EQ-5D are widely used, they may not fully capture the disease-specific burden of PsA [7, 8]. Psoriatic arthritis is a heterogeneous group of diseases characterized by involvement of different domains, including musculoskeletal involvement as well as skin involvement [9, 10]. Disease-specific instruments such as the PsAQoL offer more accurate evaluations tailored to the complex nature of PsA. Given the variability of PsA and its considerable psychological impact, a validated Turkish version of the PsAQoL is crucial for clinical treatment and research. Although it has been translated into many languages and used in many studies, Turkish validation and cultural adaptation studies have not yet been conducted [11–15].

This study aims to evaluate the Turkish validity and reliability of the PsAQoL, a disease-specific tool for assessing quality of life in patients with PsA, and to investigate its psychometric aspects.

## Methods

Patients diagnosed with PsA according to the CASPAR criteria and followed at the XXX were included in the study. Patients with severe concomitant illness were excluded and patients who signed the informed consent form were enrolled. The sample size was determined based on literature recommendations, aiming for 5 to 7 times the number of items in the scale. Accordingly, a minimum of 100 patients was planned for the 20-item questionnaire.

Permission to use and adapt the PsAQoL questionnaire into Turkish was obtained via email correspondence with S. P. McKenna et al., the developers of the original scale. The study was approved by the Ethics Committee of XXX (09.2021.62). Informed consent was obtained from individuals willing to participate.

## Patient data

Patients’ demographic, clinical, and laboratory data including age, sex, educational status, marital status, disease duration, smoking, medications, acute phase reactans incluiding C-reactive protein (CRP) and erythrocyte sedimentation rate (ESR) were recorded. Swollen and tender joint counts and the presence of peripheral arthritis, dactylitis, enthesitis, and spondylitis were evaluated.

Bath Ankylosing Spondylitis Disease Activity Index (BASDAI), Disease Activity in Psoriatic Arthritis (DAPSA), and Disease Activity Score 28 (DAS28) were used for diasese activity assessment. BASDAI is a self-report tool developed for axial involvement, consisting of six questions that assess axial and peripheral joint pain, fatigue, and morning stiffness [16]. DAPSA is also a composite score used to assess disease activity in PsA and includes the tender and swollen joints, pain, the patient’s global assessment, and C-reactive protein (CRP) levels [13]. DAS28 is based on the number of tender and swollen joints, ESR or CRP levels, and the patient’s global assessment [17].

Spinal mobility was assesed using the Bath Ankylosing Spondylitis Metrology Index (BASMI). It consists of five physical measurements: cervical rotation, tragus-to-wall distance, lumbar side flexion, lumbar flexion and intermalleolar distance [15]. Maastricht Ankylosing Spondylitis Enthesitis Score (MASES) was used to evaluate enthesitis. It assesses tenderness at 13 specific anatomical sites by palpation, providing a total score ranging from 0 to 13 [18].

Pain, fatigue, and morning stiffness severity were measured using the Visual Analog Scale (VAS). Anxiety and depression were assessed using the Hospital Anxiety and Depression Scale (HADS). This self-administered questionnaire consists of 14 items, with 7 items evaluating anxiety and 7 items evaluating depression, each scored from 0 to 3 [19]. In patients with psoriatic skin lesions, severity was evaluated via physical examination using the Psoriasis Area and Severity Index (PASI). This composite index evaluates the degree of body surface involvement and the severity of erythema, induration, and scaling in four body regions: the head, upper extremities, trunk, and lower extremities. PASI scores range from 0 to 72, with higher scores indicating more severe disease [20].

## Global health status and functional assessment

The patient’s overall health status (Patient Global Assessment, PaGA) and the physician’s global assessment (PhGA) were evaluated using the Visual Analog Scale (VAS), based on clinical and physical examination findings.

Activities of daily living were assessed using the Health Assessment Questionnaire (HAQ), which evaluates eight categories: dressing, arising, eating, walking, hygiene, reach, grip, and other activities. The questionnaire consists of 20 items scored as follows: no difficulty (= 0), some difficulty (1), much difficulty (2), and unable to do (3). The total score is divided by 20 to obtain the HAQ score. Higher scores indicate worse functional status [21].

## Assessment of general quality of life

General quality of life was assessed using the Short Form-36 (SF-36). The SF-36 health survey is the most commonly used generic quality of life instrument in medical research. It consists of 36 items grouped into eight subscales that evaluate physical and mental health: physical functioning, role limitations due to physical problems, body pain, general health, vitality, social functioning, role limitations due to emotional problems, and mental health. It also provides two summary scores: the Physical Component Summary (PCS) and the Mental Component Summary (MCS) [22–25]. The Turkish version of the SF-36 has been validated and found to be reliable [26].

## PsAQoL (psoriatic arthritis quality of life questionnaire)

The PsAQoL is a self-report, disease-specific quality of life questionnaire consisting of 20 items. Responses are given as “true” or “false,” and the total score ranges from 0 to 20, with a higher score indicating poor quality of life [6].

## Cross-cultural adaptation

The PsAQoL was translated into Turkish using the dual-panel process. Initially, the scale was translated into Turkish by two faculty members from the rheumatology clinic and an English language instructor. This Turkish version was then back-translated into English by two independent bilingual individuals who were unfamiliar with the original version of the questionnaire, and the consistency between the two versions was examined. Subsequently, the same faculty members reviewed the Turkish version, discussed the content, and made the necessary grammatical and semantic adjustments to develop a preliminary Turkish form.

In the next phase, this preliminary version was administered as a pilot test to 10 patients with PsA to evaluate the clarity of the items. The consistency and directionality of the questions were analyzed both qualitatively through expert opinion and quantitatively using statistical methods. Based on the feedback, items that patients found unclear were revised. Following these content validity procedures, the final Turkish version of the scale was established [27–29]. Items of the Turkish version of PsAQoL questionnaire are shown in ‘’Appendix’’.

## Reliability

Internal consistency was assessed to determine the reliability of the scale using Cronbach’s alpha coefficient. The alpha value ranges from 0 to 1, with values closer to 1 indicating higher internal consistency. For group comparisons, a Cronbach’s alpha value greater than 0.7 is considered acceptable [30].

## Construct validity

Construct validity (including convergent and divergent validity) was used to assess the validity of the scale. For convergent validity analysis, correlations between the PsAQoL and other established scales were examined.

## Statistical analysis

Statistical analyses were performed using SPSS version 24.0 for Windows. Descriptive statistics were presented as frequencies and percentages for categorical variables and as means, standard deviations, minimum, and maximum values for continuous variables. The One-Sample Kolmogorov–Smirnov test was used to assess the normality of the data distribution. Internal consistency analyses were conducted to evaluate the reliability of the PsAQoL which was evaluated using Cronbach’s alpha. For validity assessment, Pearson correlation analysis was used to examine the relationship between PsAQoL and other quality of life instruments. A p-value of less than 0.05 was considered statistically significant.

## Results

Of the 162 patients, 109 (67.3%) were female and 53 (32.7%) were male. The mean age of the patients was 46.3 years (SD: 11.7), and the mean disease duration was 5.8 years (SD: 6.8). The demographic and clinical characteristics of the patients are presented in Table 1.

**Table 1:** Demographic and clinical characteristics of PsA patients

Parameters	
Age, years	46.3 (11.7)
Female gender	109 (67.3%)
Level of education	
Primary school	71 (43.9%)
Middle school	20 (12.3%)
High school	43 (26.5%)
University	28 (17.2%)
Disease duration, years	5.8 (6.8)
Arthritis	80 (49.3%)
Dactylitis	10 (6.1%)
Enthesitis	68 (42%)
Axial involvement	74 (45.7%)
Tender joint count	3.8 (6.3)
Swollen joint count	0.9 (1.7)
MASES	1.7 (1.4)
VAS-pain	4.7 (2.7)
VAS-fatigue	5.5 (3)
PASI	2.9 (5.1)
DAS-28	3.1 (1.4)
DAPSA	15.2 (10.5)
BASDAI	4.5 (2.5)
BASMI	1.7 (1.4)
HAQ	0.8 (0.8)
HADS-anxiety	8.2 (4.6)
HADS-depression	7.6 (4)
SF-36 Physical component	48.5 (21.7)
SF-36 Mental component	48.4 (21.9)

Data are presented as mean (SD) or n (%)

*SF-36* Short form 36, *MASES* Maastricht Ankylosing Spondylitis Enthesitis Score, *PASI* Psoriasis Area Severity Index, *DAPSA* Disease Activity in PSoriatic Arthritis, *DAS28* Disease Activity Score 28, *BASMI* Bath Ankylosing Spondylitis Metrology Index, *BASDAI* Bath Ankylosing Spondylitis Disease Activity Index, *VAS* Visual analog scale, *HAQ* Health Assessment Questionnaire, *HADS* Hospital Anxiety and Depression Scale

## Psychometric properties of the PsAQoL

The Turkish version of the PsAQoL was found to be clear, understandable, and content-appropriate by the patients. The average time to complete the Turkish version of the PsAQoL was 3.3 ± 0.9 min. The mean PsAQoL score was calculated as 8.7 (SD:6.3) (range 0–20). The questionnaire did not exhibit floor or ceiling effects (< 15%) and there were no missing data.

## Internal consistency

The internal consistency of the Turkish version of the PsAQoL was high, with a Cronbach’s alpha coefficient of 0.930. This result indicates a strong inter-item correlation within the scale.

## Construct validity

Correlations between PsAQoL scores and both quality of life (QoL)-related and non-QoL-related indices are presented in Table 2. PsAQoL scores showed strong negative correlations with the SF-36 physical component summary (*r* = − 0.744) and mental component summary (*r* = − 0.731). Moderate negative correlations were also observed with vitality (*r* = − 0.657), role physical (*r* = − 0.640), emotional well-being (*r* = − 0.590), and pain (*r* = − 0.535). In addition, PsAQoL scores demonstrated moderate positive correlations with HAQ (*r* = 0.533), HADS-Anxiety (*r* = 0.535), and HADS-Depression (*r* = 0.517).

**Table 2:** Spearman’s correlation coefficients of PsAQoL with the other parameters for construct validity

Convergent validity		Divergent validity			
SF-36 subscales	Rho	Clinical parameters	Rho	Surveys	Rho
Physical component	− 0.744**	Age	0.150	BASDAI	0.355**
Mental component	− 0.731**	Disease duration	0.014	VAS Pain	0.408**
Physical functioning	− 0.629**	Tender joint count	0.262**	VAS Fatigue	0.447**
Physical role limitations	− 0.640**	Swollen joint count	0.170*	HAQ	0.533**
Emotional role limitations	− 0.576**	MASES	0.152	HADS Anxiety	0.535**
Vitality	− 0.657**	PASI	− 0.032	HADS Depression	0.517**
Emotional well-being	− 0.590**	DAPSA	0.409**		
Social functioning	− 0.591**	DAS28	0.322**		
Pain	− 0.535**	BASMI	0.032		
General health	− 0.536**				

*PsAQoL* Psoriatic Arthritis Quality of Life, *SF-36* Short Form 36, *MASES* Maastricht Ankylosing Spondylitis Enthesitis Score, *PASI* Psoriasis Area Severity Index, *DAPSA* Disease Activity in Psoriatic Arthritis, *DAS28* Disease Activity Score 28, *BASMI* Bath Ankylosing Spondylitis Metrology Index, *BASDAI* Bath Ankylosing Spondylitis Disease Activity Index, *VAS* Visual analog scale, *HAQ* Health Assessment Questionnaire, *HADS* Hospital Anxiety and Depression Scale

**p < 0.001, *p: 0.001–0.049

Weak or negligible correlations were observed between PsAQoL scores and variables that are not directly related to quality of life, including PASI (*r* = − 0.032), age (*r* = 0.150), and BASMI (*r* = 0.032), supporting divergent validity.

## Discussion

In recent years, there has been an increasing interest in the assessment of quality of life in rheumatic diseases. Psoriatic arthritis is a multifaceted disease that affects not only the joints and skin but also psychological well-being, functional ability, and overall quality of life [31–33]. Therefore, disease-specific and patient-reported outcome measures such as the PsAQoL are important for monitoring the disease and making treatment decisions. The current study has shown that the Turkish version of PsAQoL is a reliable and valid tool for assessing the quality of life in patients with PsA.

For PsA patients, peripheral joint pain, spinal pain, fatigue and progressive joint damage, can impair health-related quality of life and physical functioning. The dermatological manifestations of psoriasis independently contribute to quality of life impairment, affecting both physical comfort and emotional well-being. A cross-sectional multicenter study showed that both skin and joint symptoms had very strong associations with quality of life [34]. Similarly, a real-world study found that various PsA manifestations, including dactylitis, enthesitis, peripheral arthritis, and axial disease, were each independently associated with reduced quality of life [35]. The impact of these various domains varies in different populations. In a global survey, musculoskeletal system symptoms (especially joint pain, joint tenderness, joint swelling, stiffness, inflammatory back pain, and enthesitis) and skin involvement are more common in the US compared to other countries, while social embarrassment or disapproval is more commonly reported in Spain, and fatigue is most prevalent among patients in France [36]. Our findings, in addition to their validity, highlight the clinical importance of PsAQoL in capturing the multidimensional burden of PsA in Turkish patients. Especially, the relationships observed with both physical and emotional health parameters highlight the need for holistic, patient-centered care models in the management of PsA.

The PSAQOL questionnaire was developed in 2004 to assess disease-specific quality of life for PSA and has been widely accepted [6]. Many studies have been reported concerning the reliability and validity of the PSAQOL questionnaire in various languages [11–14]. The present study successfully translated the PSAQOL into Turkish and showed that the Turkish version of the PSAQOL was easily understood by patients. In addition, with its short structure (20 items) and binary response format, the survey is practical for daily clinical practice.

The Cronbach’s alpha value evaluated for the psychometric performance of the Turkish version of PsAQoL was found to be 0.93. These findings are strongly supported by similar results in validations in other languages; the Dutch version reported a Cronbach’s alpha value of 0.92 in a sample of 211 patients [11]. The German version similarly demonstrated strong internal consistency with a 0.92 alpha in a multicenter cohort [14]. Similarly, the Portuguese version found a Cronbach’s alpha value of 0.91 [13]. These similar values among the different cultural adaptations highlight the reliability and generalizability of PsAQoL as a disease-specific tool for measuring quality of life in PsA patients. The similarity in psychometric results also supports the structural integrity of the original questionnaire and its effectiveness in capturing patient-reported outcomes across different populations.

Structural validity has also been supported by the strong correlations between the PsAQoL and the SF-36 subscales that assess quality of life. The highest correlations were observed with the physical component and the mental component indicating that PsAQoL comprehensively reflects both the physical and psychosocial aspects of the disease’s impact. Additionally, moderate correlations with HAQ and HADS (both anxiety and depression) confirm its relationship with functional status and emotional health, which is consistent with previous studies [5, 7, 37]. Importantly, weak correlations with unrelated clinical parameters (e.g., age, PASI, MASES) support the scale’s discriminative validity.

In the Dutch validation study, the impact of psoriasis was evaluated not with an objective clinical severity index like the PASI score but with the Skindex-17 questionnaire, which shows dermatological symptoms reported by the patient and their psychosocial consequences. In contrast, we evaluated psoriasis severity using an objective clinical tool like PASI and observed a weak correlation with PsAQoL. The patient-reported skin-related burden captured by Skindex-17 appears to be more closely associated with PsAQoL, while objective disease severity scores like PASI may not fully reflect the patient’s subjective experience. These findings further support the distinct validity of PsAQoL in our cohort [11].

The Turkish version of the PsAQoL was found to be easy to understand and quick to complete, with a mean completion time of 3.3 ± 0.9 min, confirming its usability in both clinical and research settings​. This result is consistent with earlier validation studies conducted in other languages. The Dutch version reported a similar mean completion time of approximately 4 min, while the Portuguese version was completed on average in 5.7 min and the German version in about 4.7 min, all supporting the instrument’s low respondent burden [11, 13, 14]​. Additionally, the Turkish version demonstrated no floor or ceiling effects (both < 15%) and had zero missing responses.

In conclusion, the Turkish version of the PsAQoL was found to possess strong psychometric properties, confirming its validity and reliability as a disease-specific instrument for assessing quality of life in PsA. It’s clear structure and ease of administration make it highly suitable for use in both routine clinical practice and research settings. Moreover, this study contributes to the existing literature as the first to validate the PsAQoL in a Turkish population, thereby supporting its broader cross-cultural applicability.

## Electronic supplementary material

Below is the link to the electronic supplementary material.

Supplementary material 1 (DOCX 16 kb)
