# Supplementary material for: Validity and psychometric characteristics of the psoriatic arthritis quality of life (PSAQoL) questionnaire in the Turkish population
Source: Rheumatol Int. 2025 Jun 16;45(7):155. doi: 10.1007/s00296-025-05911-6 (PMC12170716; doi:10.1007/s00296-025-05911-6)
Supplement: Supplementary file 1 — Supplementary material 1 (DOCX 16 kb) [file 296_2025_5911_MOESM1_ESM.docx]

**Psoriatik Artrit Yaşam Kalitesi Anketi (PSAQoL)**

Lütfen her soruyu dikkatlice okuyunuz ve sizin **şu andaki** durumunuza uygun olup olmadığına karar veriniz

Doğru Yanlış

1. Ne yapsam yoruluyorum. ☐ ☐
2. Bütün vücudumu yıkamakta zorluk çekiyorum . ☐ ☐
3. Dışarı çıkmak ve insanlarla birlikte olmak için çok çaba harcamam gerekiyor. ☐ ☐
4. Hayatımın hiç tadı olmadığını düşünüyorum. ☐ ☐
5. Günden güne bağımsızlığımı kaybettiğimi düşünüyorum. ☐ ☐
6. Sık sık kendime öfkeleniyorum. ☐ ☐
7. Yapmak istediğim şeyleri yapamıyorum. ☐ ☐
8. Kendimi olduğumdan daha yaşlı hissediyorum. ☐ ☐
9. Ailem veya arkadaşlarımla birlikte etkinliklere katılmam çok zor oluyor. ☐ ☐
10. Hastalığım gidebileceğim yerleri kısıtlıyor. ☐ ☐
11. Bir şeyler yapabilmem için kendimi zorlamam gerekiyor. ☐ ☐
12. Diğer insanlardan kolayca rahatsız oluyorum. ☐ ☐
13. Bir iş yaparken dinlenmek için sık sık ara veriyorum. ☐ ☐
14. Kendimi başkalarına bağımlı hissediyorum. ☐ ☐
15. Sabahları kendimi toparlayıp güne başlamam uzun süre alıyor. ☐ ☐
16. Hastalığımın acısını yakınlarımdan çıkarıyorum. ☐ ☐
17. Anlık kararlarla iş yapamıyorum. ☐ ☐
18. Kendimi evime hapsedilmiş gibi hissediyorum. ☐ ☐
19. Günlük işlerimi kısıtlamam gerekiyor. ☐ ☐
20. Hastalığım kişisel ilişkilerimde zorluk çıkarıyor. ☐ ☐
